# Effects of vitamin D supplementation on alveolar macrophage gene expression: preliminary results of a randomized, controlled trial

**DOI:** 10.1186/2049-6958-9-18

**Published:** 2014-03-26

**Authors:** Alicia K Gerke, Alejandro A Pezzulo, Fan Tang, Joseph E Cavanaugh, Thomas B Bair, Emily Phillips, Linda S Powers, Martha M Monick

**Affiliations:** 1 Department of Internal Medicine, University of Iowa, 200 Hawkins Dr, Iowa City 52242, IA, USA; 2 Department of Biostatistics, University of Iowa, 105 River Street, Iowa City 52242, IA, USA; 3 University of Iowa Institute for Human Genetics, Iowa City, IA, USA

**Keywords:** Alveolar, Gene expression, Immunity, Innate, Macrophages, Randomized controlled, Vitamin D

## Abstract

**Background:**

Vitamin D deficiency has been implicated as a factor in a number of infectious and inflammatory lung diseases. In the lung, alveolar macrophages play a key role in inflammation and defense of infection, but there are little data exploring the immunomodulatory effects of vitamin D on innate lung immunity in humans. The objective of this study was to determine the effects of vitamin D supplementation on gene expression of alveolar macrophages.

**Methods:**

We performed a parallel, double-blind, placebo-controlled, randomized trial to determine the effects of vitamin D on alveolar macrophage gene expression. Vitamin D3 (1000 international units/day) or placebo was administered to adults for three months. Bronchoscopy was performed pre- and post-intervention to obtain alveolar macrophages. Messenger RNA was isolated from the macrophages and subjected to whole genome exon array analysis. The primary outcome was differential gene expression of the alveolar macrophage in response to vitamin D supplementation. Specific genes underwent validation by polymerase chain reaction methods.

**Results:**

Fifty-eight subjects were randomized to vitamin D (n = 28) or placebo (n = 30). There was a marginal overall difference between treatment group and placebo group in the change of 25-hydroxyvitaminD levels (4.43 ng/ml vs. 0.2 ng/ml, p = 0.10). Whole genome exon array analysis revealed differential gene expression associated with change in serum vitamin D levels in the treated group. CCL8/MCP-2 was the top-regulated cytokine gene and was further validated.

**Conclusions:**

Although only a non-significant increased trend was seen in serum vitamin D levels, subjects treated with vitamin D supplementation had immune-related differential gene expression in alveolar macrophages.

**Trial registration:**

ClinicalTrials.org: NCT01967628.

## Background

Vitamin D is suspected to be an important factor in susceptibility to bacterial and viral infections of the lung [1]. Historically, Vitamin D has been used as a treatment for tuberculosis [2]. More recently, deficiency has been associated with influenza, increased severity of community acquired pneumonia and development of chronic obstructive pulmonary disease (COPD) [3-5]. The mechanisms behind these effects are unclear, but may reflect, in part, the effects of vitamin D on the innate immune response of the lung.

Prior studies have indicated that vitamin D plays an important intracrine role in serum monocyte and macrophage response to infection [6]. Macrophages, monocytes, T-cells, and dendritic cells express 1-alpha-hydroxylase which converts 25-hydroxyvitamin D (25(OH)D) to its active form 1,25-dihydroxyvitamin D (1,25(OH)D). Macrophages constitutively express the vitamin D receptor and serum macrophages have been shown to enhance innate immunity by vitamin D regulated production of cathelicidin in response to mycobacteria [7]. Vitamin D-regulated human cathelicidin production also promotes autophagy of the macrophage, an important process in removing infectious antigens [8].

Despite these reports of the importance of vitamin D in serum macrophages, there are few data regarding the specific relationship of the alveolar macrophage to vitamin D status or supplementation. In this study, the objective was to determine how vitamin D regulates lung innate immunity. We performed a double-blind, placebo controlled, randomized trial in humans to investigate the effects of vitamin D on alveolar macrophage gene expression. The hypothesis was that vitamin D supplementation will affect the gene expression profile of the alveolar macrophage in human subjects.

## Methods

### Trial design

This study was performed as a specific analysis of a double-blind, parallel-group, randomized controlled intervention study of the effects vitamin D on alveolar macrophage gene expression in both smoking and nonsmoking subjects. The study was approved by the institutional review board at the University of Iowa (IRB# 200607708) and written consent was obtained from all subjects. The study is registered on ClinicalTrials.org (NCT01967628). An independent data safety monitoring board met regularly to review safety and occurrence of adverse events. The study was monitored by the sponsoring agency to evaluate study progress, quality of data collection, and adherence to regulatory and study protocols.

### Participants

Subjects were included if they were 18–60 years old and able to understand and sign a consent form. Subjects were recruited from the community via advertisements and word-of-mouth. Subjects were excluded if they had taken a multivitamin or vitamin D supplement within the previous three months, were pregnant or breastfeeding, had a vaccination within one month, had a history of asthma, diabetes, heart disease, allergy to lidocaine, or any other medical problem that would increase risk of bronchoscopy (e.g. renal disease or electrolyte imbalance). Subjects were also excluded if they had a respiratory infection in the six weeks prior to enrollment, history of pneumonia within three years, history of positive tuberculin skin test, or use of antibiotics for any purpose within six weeks of enrollment. Subjects were excluded if they were taking any prescription medication except for hormonal birth control, topical medications for mild skin ailments, selected antidepressants, levothyroxine, as needed acid reflux treatment, as needed over-the-counter antihistamine, or as needed sleep aids.

### Intervention

After obtaining informed consent, included subjects underwent randomization to 1000 international units (IU) per day of cholecalciferol (Vitamin D3) by oral capsule or a matching placebo capsule for a three-month period of time (total dose of 90,000 IU). Prior to treatment, each subject underwent bronchoscopy with bronchoalveolar lavage to obtain alveolar macrophages. After three months of treatment with vitamin D or placebo, each subject then underwent a second bronchoscopy to obtain alveolar macrophages. Bronchoscopies were performed by a pulmonary physician in standard fashion using a flexible bronchoscope (model P160 or P180; Olympus) at the University of Iowa Hospitals and Clinics (Iowa City, Iowa, USA) between May 2008 and July 2010. Subjects were premedicated with either morphine 10 milligrams (mg) or meperidine 12.5-25 mg intramuscularly and atropine 0.6 mg intramuscularly prior to bronchoscopy. Subjects did have the option of foregoing premedication. Subjects were then administered topical anesthesia with 2%-4% lidocaine to numb the airway. Under standard clinical monitoring, the bronchoscope was introduced trans-nasally or transorally into the right lung. To collect alveolar macrophages, bronchoalveolar lavage was performed by instilling five aliquots of 20 milliliters of sterile normal saline into three segments of the lung and suctioning back into a trap container. Blood was drawn at each subject visit prior to bronchoscopy to assess for levels of 25(OH)D, 1,25(OH)D, parathyroid hormone, calcium, and creatinine. Women of childbearing potential underwent urine pregnancy screen prior to each bronchoscopy.

### Outcomes

The primary outcome was to assess the effects of vitamin D supplementation on differential gene expression as determined by microarray analysis of alveolar macrophage messenger RNA (mRNA). Gene expression was compared between placebo and treatment groups, as well as a paired analysis pre- and post-supplementation within the group treated with vitamin D.

### Randomization

Subjects were randomized on a 1:1 basis to Vitamin D3 or a matching placebo capsule by a computer generated randomization list provided by the data coordinating center. Each study participant was assigned to a study product allocation number by the data coordinating center after study consent. Study participants, clinical research team, sponsoring agency, and data analysts were blinded to intervention assignment. The vitamin D and placebo were identical in appearance.

### Statistical analysis: clinical data

All clinical data analysis was conducted using SAS version 9.1.3 (SAS Institute, Inc., Cary, North Carolina). To confirm whether the randomization scheme worked in our study, the two sample *T*-test was used to compare whether there were differences in two treatment groups for all the continuous demographic variables. The *T*-test was also used to investigate between-group differences in clinical outcomes. The Fisher’s Exact test was used for gender to test whether the assignment was balanced.

### Preparation of RNA and microarray analysis

RNA preparation, quality analysis, and microarray analysis were done as previously described [9]. Measurements of genome-wide macrophage mRNA expression were conducted using the GeneChip Human Exon 1.0 ST Arrays (Affymetrix). Microarray data were analyzed with Partek Genomics Suite, version 6.5, software (Partek, St. Louis, MO, USA). The data were assessed for quality and subjected to robust multi-array averaging normalization. The method of statistical analysis was the paired *T*-test (before vitamin D supplementation versus after vitamin D supplementation in the treated group). A p of 10^−5^ was considered significant in this analysis. Gene pathway analysis was conducted using the GoMiner suite (Genomics and Bioinformatics Group, Bethesda, MD, USA) of algorithms. The expression data has been deposited in NCBI Geo repository.

### Gene validation and analysis

Three candidate genes, chosen for biological plausibility and statistical significance, were validated by polymerase chain reaction (PCR). The genes, CCL8, P2RY10, and SOX13 were analyzed in samples from subjects whose serum 25(OH)D levels rose greater than 5 ng/ml. Total RNA (300 ng) was reverse-transcribed to cDNA using iScript cDNA Synthesis kit (Bio-Rad). SYBR Green-based quantitative PCR reactions (BioRad) were performed as previously described [9]. Specificity of the amplification was confirmed using melting curve analysis. Expression levels were defined as a ratio between the threshold cycle (Ct) values of CCL8, P2RY10, SOX13 and the endogenous control, HPRT.

## Results

### Overall distribution of subjects

**Figure 1:**
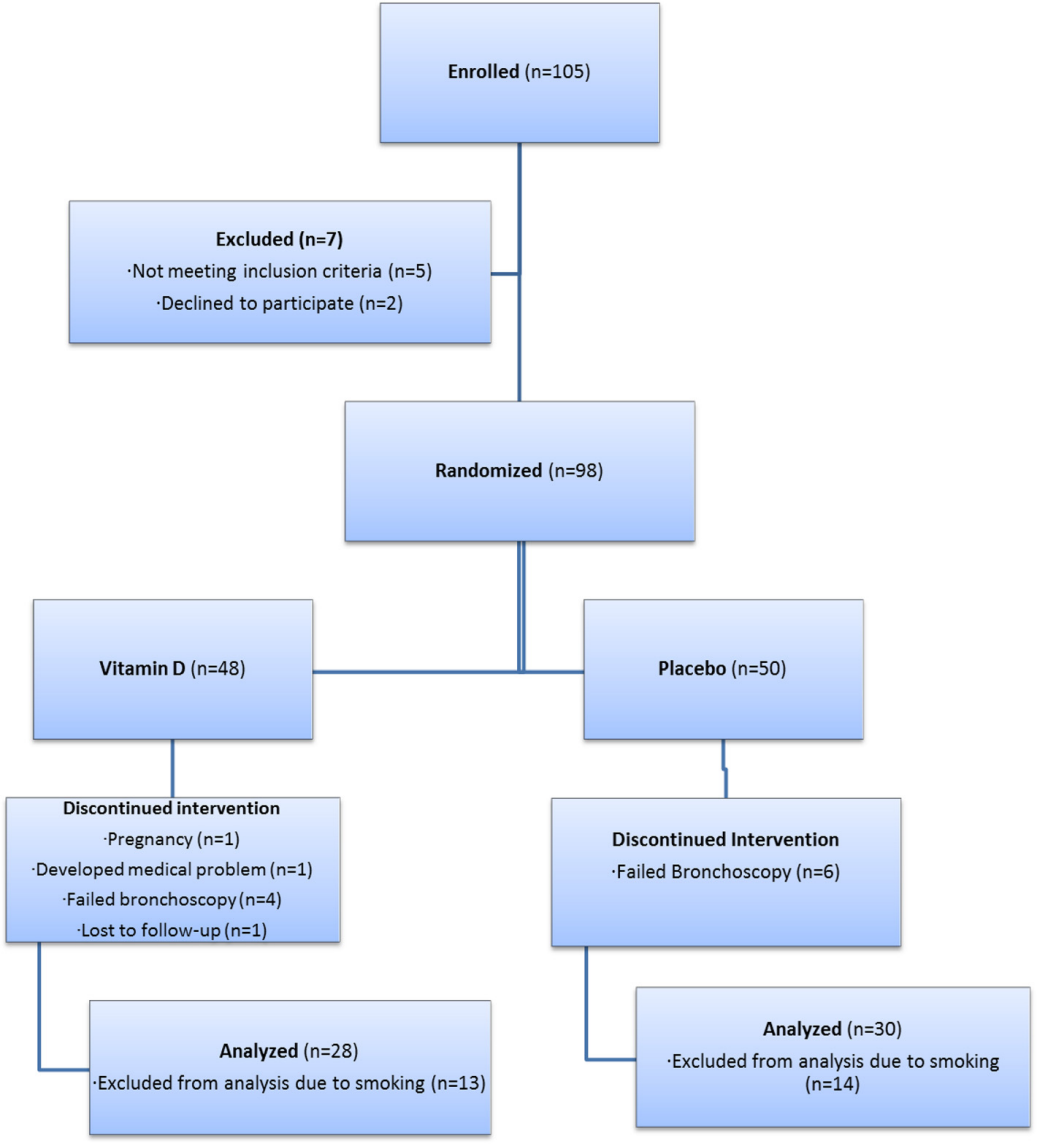
Subjects enrollment.

105 human subjects were enrolled (Figure 1) between May 2008 and July 2010. The trial was completed after a predetermined endpoint of time was achieved. After signing informed consent, seven of the enrolled subjects were found to have exclusion criteria and were not randomized. Ninety-eight subjects were randomized to intervention. Thirteen subjects were terminated early. For the purposes of this analysis, we excluded 27 subjects who smoked from the analysis, as smoking has known profound effects on macrophage gene expression. For the final analysis, 58 subjects were included.

### Clinical outcomes

**Figure 2:**
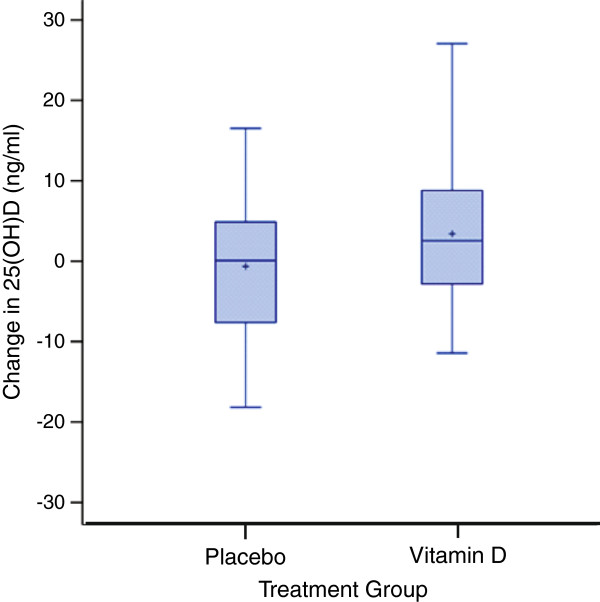
Treatment effect for 25-hydroxyvitamin D (25(OH)D) levels by treatment group (vitamin D versus placebo) (p = 0.10).

Demographics and labs, stratified by treatment group (Vitamin D versus Placebo), of the 58 subjects who were analyzed are described in Table 1. There were no differences in age or lab values between treated and placebo groups, indicating that randomization was effective. There were marginal overall differences between treatment and placebo groups in the change of 25(OH)D level (p-value = 0.10) and parathyroid hormone level (p = 0.05), but no significant changes in 1,25(OH)D levels or calcium levels (Table 2). Figure 2 shows the distribution of the change of 25(OH)D levels by treatment group.

**Table 1:** Comparison of patient’s characteristics by treatment group: vitamin D treatment versus placebo

**Variable**	**Placebo group**	**Treatment group**	**P**
Age (years)	26.3	25.6	0.72
Baseline 25(OH)D^*^ level (ng/ml)	32.9	30.9	0.56
Baseline 1,25(OH)D^†^ level (pg/ml)	49.3	51.2	0.67
Baseline parathyroid hormone (pg/ml)	34.9	36.8	0.57
Baseline calcium level (mg/dl)	9.2	9.1	0.47
Male gender (%)	36.7%	63.3%	0.20

*25-hydroxyvitamin D.

^†^1,25-hydroxyvitamin D.

**Table 2:** Treatment effect of vitamin D supplementation

**Variable**	**Placebo group**	**Treatment group**	**P**
Change in 25(OH)D^*^ level (ng/ml)	0.20	4.43	0.10
Change in 1,25(OH)D^†^ level (pg/ml)	1.13	−3.71	0.30
Change in parathyroid hormone (pg/ml)	7.83	1.21	0.05
Change in calcium level (mg/dl)	−0.10	0.01	0.24

*25-hydroxyvitamin D.

^†^1,25-hydroxyvitamin D.

### Gene expression

Paired analysis was performed on the group treated with vitamin D (pre- and post-supplementation) (Table 3). There were seven genes with P less than 10^5^: Putative P2Y purinoceptor 10 (NM_014499), monocyte chemoattractant protein 2 (CCL8/MCP-2)(NM_005623), Homer protein homolog 2 (NM_199330), asparaginyl-tRNAsynthetase 2 (NM_024678), Ras association (RalGDS/AF-6) domain family member (NM_014737), SRY (Sex Determining Region Y)-Box 13 (NM_005686), Melanocortin 3 receptor (NM_019888). Only the monocyte chemoattractant protein 2 (CCL8/MCP-2) had a fold-change of greater than 1.5. Pathway analysis showed differentially regulated immune pathways, but all had a false discovery rate of greater than 0.25 (Table 4). There were no significant differences in alveolar macrophage gene expression between treated and placebo groups.

**Table 3:** The thirty most differentially regulated genes in subjects treated with vitamin D supplementation (pre- versus post-supplementation)

**RefSeq**	**Gene symbol**	**P**	**Fold change**
NM_014499	P2RY10	1.45E-05	1.30
NM_005623	CCL8	1.99E-05	1.51
NM_199330	HOMER2	2.11E-05	1.25
NM_024678	NARS2	2.16E-05	1.23
NM_014737	RASSF2	2.86E-05	1.15
NM_005686	SOX13	8.37E-05	−1.12
NM_019888	MC3R	9.95E-05	1.41
NM_173079	RUNDC1	0.00017	1.97
NM_000693	ALDH1A3	0.00019	1.22
NM_024839	RPP21	0.00022	−1.35
NM_198562	C3orf62	0.00022	−1.21
NM_005164	ABCD2	0.00027	1.16
NM_017649	CNNM2	0.00028	1.19
NM_014240	LIMD1	0.00028	−1.18
NM_004123	GIP	0.00030	1.27
NM_001040653	ZXDC	0.00031	−1.20
NM_001100389	TMEM192	0.00033	−1.14
NM_207111	RNF216	0.00033	−1.18
NM_014033	METTL7A	0.00035	1.18
NM_014751	MTSS1	0.00036	1.14
NM_002145	HOXB2	0.00037	1.20
NM_144669	GLT1D1	0.00037	1.20
AF258559	LYRM4	0.00040	−1.79
NM_002258	KLRB1	0.00046	1.28
NM_145178	ATOH7	0.00048	1.22
NM_006545	NPRL2	0.00048	−1.11
NM_015550	OSBPL3	0.00048	−1.14
NM_003239	TGFB3	0.00051	1.18
NM_001719	BMP7	0.00054	1.17
AY204749	NCRNA00114	0.00055	1.27

**Table 4:** Top differentially regulated gene pathways in treated subjects pre- versus post-supplementation*

**Differentially regulated gene pathways**	**Size**	**Enrichment score**	**Normalized enrichment score**
Insulin growth factor-1/mTOR pathway	20	0.59	1.41
Calcineurin pathway	18	0.57	1.35
Rac1 pathway	22	0.55	1.32
Interleukin-10 anti-inflammatory pathway	17	0.56	1.30
B-cell receptor pathway	33	0.51	1.28
Cytokine pathway	21	0.53	1.27
Caspase pathway	23	0.52	1.27

*All pathways have false discovery rate of greater than 0.25.

### Gene validation

**Figure 3:**
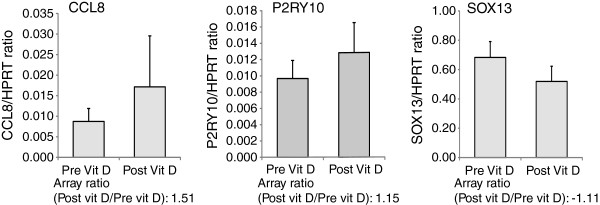
Comparison of pre and post supplementation mRNA of candidate genes in subjects treated with vitamin D whose 25-hydroxyvitamin D level rose greater than 5 ng/ml (measured by polymerase chain reaction).

To validate the array data we chose three genes based on plausibility and significance (two upward trending, and one downward trending) in the paired analysis of the treated group. We found that with all three genes, the individual assays (n = 11) replicated the change trends found in the array data (Figure 3).

### Safety and adverse events

There were fourteen total adverse events reported among eight of the subjects: nine events in placebo group and five events in the vitamin D treated group. There were no serious adverse events or procedure-related adverse events among the 58 subjects. Indigestion after taking the study drug was the only study-related adverse event in a patient taking vitamin D. Adverse events determined to be unrelated to the study included epididymitis, urinary tract infection, increased worry, irritability and/or depression, complication of an unrelated medical procedure during the study period, abnormal vaginal bleeding, uterine cramping, and head injury.

### Compliance

In the treatment group, thirteen subjects (46%) completed full therapy, and eleven (39%) of subjects had residual doses. Among those that did not take full dose, the average number of missed capsules was 6.5 capsules (range 1–15), equating to an average total missed dose of 6500 IU. Four treated subjects did not bring back their pill bottles for compliance analysis. In the placebo group, thirteen subjects completed full therapy (43%) and sixteen subjects (53%) had missed doses. The average number of missed capsules was 8.8 (range 1–30). One placebo subject did not bring back the pill bottle for analysis.

## Discussion

In this study, we used microarray technology to determine gene expression differences in human subjects treated with vitamin D supplementation. Although the results were not statistically strong, trends show that there are small differences in resting alveolar macrophage gene expression modulated by vitamin D supplementation. To our knowledge, this is the first study that shows the effects of vitamin D supplementation on alveolar macrophage gene expression in a clinical trial in humans.

The importance of vitamin D in bone health is well-established and has been the standard by which adequate serum levels and supplementation have been based. The effects on the non-classical roles of vitamin D in autoimmune disease, cancer, and immunity have sparked intense controversy over the past decade. It is unknown whether higher recommended daily doses are needed, or what the ‘normal’ 25(OH)D level should be. In immunity, the question of whether vitamin D effects are primarily anti-inflammatory or antimicrobial is unclear, and data regarding its effects supporting both innate and adaptive immunity exist [10,11]. For innate immunity, vitamin D has been shown to enhance the immune response to infection by increasing cathelicidin and phagocytosis in serum macrophages, important mechanisms in fighting infection [7]. Since alveolar macrophages and airway epithelial cells are able to convert vitamin D to its active form, it is plausible that this type of antimicrobial action is also present in the lung [12]. However, to date, randomized controlled trials on whether vitamin D supplementation decreases the incidence of respiratory infections have been conflicting in various populations [13-15].

Our strongest upregulated inflammatory gene is monocyte chemoattractant protein 2 (CCL8/MCP-2). CCL8 is a chemokine involved in attracting human leukocytes, lymphocytes, monocytes, natural killer cells, eosinophils, and basophils via a number of receptors. It is also thought to be a potent inhibitor of HIV through through its action on the CCL5 (chemokine ligand 5) [16]. CCL8 has been found to be elevated in tuberculosis infection and proposed as a potential biomarker of tuberculous pleural effusions [17-19]. Prior studies have indicated effects of vitamin D on suppression of MCP-1 and inflammation, but there have been no prior reported studies that we are aware showing a relationship between vitamin D and CCL8/MCP-2 [20,21]. Given the importance of CCL8 in anti-viral and anti-tuberculous activity, this cytokine warrants further study in relation to vitamin D and the lung.

There are a number of factors that may have affected our results towards a less robust gene expression analysis. First of all, despite good compliance with treatment, the amount of vitamin D supplementation was not enough to uniformly raise serum 25(OH)D levels in all of our subjects, and thereby, likely diluted the true effects of vitamin D. This may be similar to prior study showing no difference in outcomes in subjects with tuberculosis treated with vitamin D; however, the treated patients did not show an increase in their vitamin D levels [22]. In our validation process, we tested the gene expression of the candidate genes in the patients who increased their 25(OH)D levels and were able to show a more pronounced effect. Paired analysis of the treated group showed more significant differences in gene expression, as it likely reduced the baseline subject variability. Another limitation is that the alveolar macrophages were harvested in steady state without stimulation by antigens or microbes that may be needed to promote vitamin D-regulated transcription. Next, our subject population was relatively young, limiting generalizability to older or elderly populations. Lastly, our sample size may have not been large enough, with high variability within groups, to provide power to this type of complex whole genome analysis. Future research is warranted with clinical trials with further refinement of patient groups, increased sample size, and protocol methods to ensure enough vitamin D to raise serum vitamin D levels. Given the number of vitamin D insufficient or deficient people in the population, research on vitamin D’s effects on immunity could have important public health implications.

## Conclusions

In conclusion, we found small, but potentially important, differences in gene expression of the resting alveolar macrophage in human subjects supplemented with vitamin D. The results of this study, both of the microarray analysis and the lessons learned in the trial methods, will help design future studies to evaluate the effects of vitamin D on lung innate immunity. Future studies need to focus on the correlation of effects with serum 25(OH)D levels, the response of activated alveolar macrophages to vitamin D, and effects related to gender, age, and race.

## Availability of supporting data

The data sets supporting the results of this article are available in the National Center for Biotechnology Information (NCBI) gene expression omnibus (GEO) repository: accession number GSE56583, http://www.ncbi.nlm.nih.gov/geo/query/acc.cgi?acc=GSE56583.

## Abbreviations

25(OH)D: 25-hydroxyvitamin D; 1,25(OH)D: 1,25- hydroxyvitamin D; CCL5: Chemokine ligand 5; CCL8: Chemokine ligand 8; IU: International units; MCP: Monocyte chemoattractant protein; Mg: Milligrams; mRNA: Messenger RNA; PCR: Polymerase chain reaction.

## Competing interests

The authors declare that they have no competing interests.

## Authors’ contributions

AKG, the guarantor of the manuscript, contributed to the design of the study, was responsible for data collection and analyses, and contributed to the writing and revising all drafts of the manuscript. AAP was responsible for data analyses, and contributed to the writing and revising of the manuscript. FT was responsible for data analyses, and contributed to writing and revising of the manuscript. TBB was responsible for data analyses, and contributed to revising the manuscript. JEC contributed to data analyses, and to the writing and revising of the manuscript. EP was responsible for study implementation, data collection, analysis and revising of the manuscript. LSP contributed to data collection, analyses, and to the writing and revising of the manuscript. MMM contributed to the original design of the study, data collection and analyses, and to the writing and revising of the first draft and subsequent drafts of the manuscript. All authors contributed to final approval of the manuscript.
